# NCI Awardee Skills Development Consortium (NASDC): a Successful Novel Educational Effort to Maintain Early Academic Competitiveness: PART 1—Overview of the NASDC Coordinating Center

**DOI:** 10.1007/s13187-025-02635-w

**Published:** 2025-06-10

**Authors:** Claire F. Verschraegen, Mia Hashibe, Ushma S. Neill, William Pirl, Elizabeth Olson Hexner, Ann O’Connell, NASDC Consortium

**Affiliations:** 1 Division of Medical Oncology, Department of Internal Medicine, The Ohio State University Comprehensive Cancer Center, 1800 Cannon Drive, Lincoln Tower 1330, Columbus, OH 43210 USA; 2 Division of Public Health, Department of Family and Preventive Medicine, University of Utah School of Medicine, Salt Lake City, UT USA; 3 Office of Scientific Education and Training, Memorial Sloan Kettering Cancer Center, New York, NY USA; 4 Department of Supportive Oncology, Dana-Farber Cancer Institute, Boston, MA USA; 5 Abramson Cancer Center, University of Pennsylvania, Philadelphia, PA USA

**Keywords:** NCI Awardee Skills Development Consortium, NASDC, Skills Development, NCI-funded Junior Investigators, Evaluation, Mentorship, Leadership, Resilience, Cell and Gene Therapy, Immuno-Oncology, Health Disparities, Program Evaluation

## Abstract

The pilot National Cancer Institute (NCI) Awardee Skills Development Consortium (NASDC) was designed and funded by the NCI in 2019 to support a group of multi-institutional educational projects to help early-career NCI grantees remain academically competitive. Retention and academic competitiveness of early-career awardees are critical for advancing cancer research; therefore, NASDC sought to develop the next generation of leaders to meet the nation’s biomedical, behavioral, and clinical cancer research needs. NASDC comprised a group of institutions supported through cooperative agreement awards issued by the NCI, including a U24 coordinating center (U24CC) to support logistics and evaluations and four institutional awardees to deliver evidence-based scientific and educational content (UE5 awards for research education projects [UE5]). The U24CC managed applications, conducted course evaluations, and facilitated the consortium’s administrative structure. The consortium infrastructure consisted of a Governance Steering Committee with five working groups to manage the projects. A web portal was developed to help recruit eligible applicants to attend the courses. Each course was delivered five to six times over 3 years, and an evaluation system was implemented to assess outcomes within courses and across courses and cohorts. Originally intended to be in-person short courses, the COVID pandemic forced the projects to adapt to virtual platforms. NASDC aimed to build a stable pool of leading cancer researchers. NASDC successfully supported junior faculty NCI grantees by delivering evidence-based educational content, despite the COVID-19 pandemic. The intent of the program was implemented smoothly, and courses were delivered effectively. Although NCI did not reissue the program in 2021 for budgetary reasons, some of these courses are now funded by an R25 mechanism. This is the first in a series of papers detailing this program’s outcomes.

## Introduction

In 2016, the US Congress established the Next Generation Researchers Initiative (NGRI), as part of the 21 st Century Cures Act (Title II, Subtitle C: Supporting Young Emerging Scientists), to promote opportunities for new researchers and earlier research independence. Examples of these efforts include policies to increase funding opportunities, training, and mentorship programs and enhancing workforce diversity for new researchers. Skills that are required for success include the ability to stay current with new technologies, write competitive grant applications, manage personnel and finances of a research program, build a trusted network of collaborators, provide mentorship to the next generation of researchers, and remain up to date with current and relevant technological advances in their field.

Teaching these skills is lacking in typical graduate/medical student academic programs, as they often do not fit neatly into standard curricula, and postdoctoral/fellowship training does not provide sufficient educational content in many of these areas. The National Cancer Institute (NCI) wanted to maximize the outcomes from its investment in the junior faculty it funded and help them continue their early-career funding successes [1]. In 2019, two requests for applications (RFA-CA-19–010 and RFA-CA-19–011) were published to fund institutes to test the hypothesis that specialized training in leadership and newer technologies might grow the academic careers of NCI-funded junior faculty (the NCI Awardee Skills Development Consortium [NASDC] clientele). To accomplish this, a new consortium was established.

### The NCI Awardee Skills Development Consortium and NCI Requirements

**Fig. 1:**
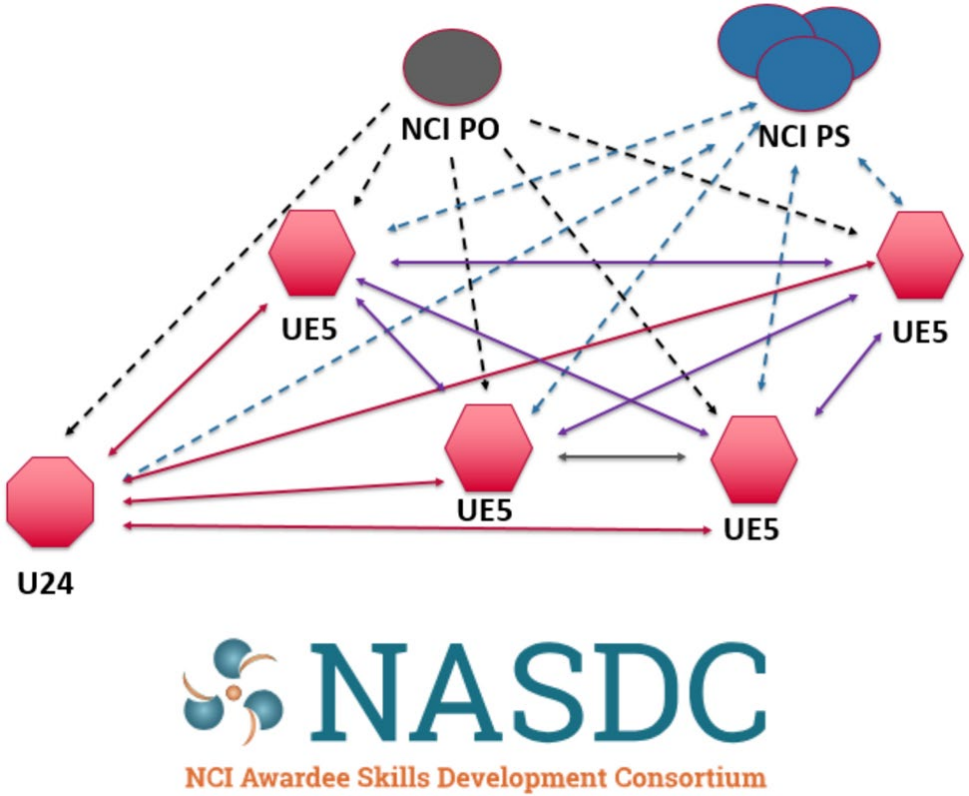
NASDC network, relationships, and branding. NCI PO, National Cancer Institute Program Officer; NCI PS, National Cancer Institute Project Scientist; UE5, Each Individual Course Center; U24, Coordinating Center

In 2020, the NASDC was funded through an award (U24) for the coordinating center and four institutional awards (UE5 s) for short educational courses complementing other formal training programs and enhancing the National Institutes of Health’s (NIH’s) national efforts in biomedical, behavioral, and clinical research. NASDC’s goals were to provide opportunities for current NCI junior faculty grantees (assistant professors, instructors, research scientists, or equivalent) to enhance their skills in areas critical for establishing and maintaining successful independent academic cancer research careers and to develop the next generation of research leaders. Each of the four UE5 awards had a specific course focus: Harvard Medical School coached leadership and resilience skills; Memorial Sloan Kettering (MSK) taught immuno-oncology; University of Pennsylvania (UPenn) delivered “the cell and gene therapy toolkit”; and University of Utah (Utah) focused on leadership, mentorship, and health disparities. A Steering Committee (SC) was established to provide overall governance and oversight to the whole program, ensuring adherence to the consortium’s objectives, timelines, and quality standards, while also offering strategic direction and addressing any challenges that arose throughout the program. The Ohio State University (OSU) was awarded a U24 Coordinating Center (U24 CC) grant to build the infrastructure that supported the delivery of the four UE5 courses, including logistics, communication, and collection of data to review outcomes among five educational domains: (1) recruitment, (2) curriculum, (3) evaluations, (4) online portal, and (5) publications. NASDC branding (Fig. 1) and the web portal engaged applicants, allowing them to complete applications, submit evaluations, and retrieve course curricula. The U24 CC actively worked with the NCI Project Scientists to identify the target population for whom NASDC was created. The consortium was formed as the COVID-19 pandemic began and vaccination was not yet available; the original plans for in-person meetings/courses were adapted to a virtual environment instead [2]. Herein, we will discuss the creation of the NASDC infrastructure. Future papers in this NASDC series will present additional outcomes of the consortium.

## Methodology

### NASDC Governance

The SC governed NASDC and had representation from each UE5 awardee institution, the U24 CC, and NCI. The SC designated five working groups (WGs) to manage the five NASDC-specific educational domains were outlined above. The U24 CC administered and maintained SC and WG records. Meetings took place regularly by Zoom to discuss the following elements:

1. Review of meeting minutes
2. Advertisement of NASDC short courses
3. Participant recruitment
4. Logistic support during course implementation
5. Arrangements for NASDC teleconferences and an annual SC in-person meeting
6. Evaluation of NASDC courses and the overall NASDC program

Tasks to accomplish were identification of the prospective learners, advertisement, recruitment, application triages, data collection, course scheduling, creation of a web portal, evaluations, and publication of results. The activity list was itemized, and an operations plan was drafted to coordinate the appropriate NASDC infrastructure. Modern business techniques increased the efficiency and accountability of NASDC management and were approved by the SC for the creation of forms, timelines, benchmarks, logistic support, and evaluations.

### NCI’s Role

The NCI played a pivotal role in conceptualizing and issuing the program announcement and used a cooperative agreement for the funding and administration of NASDC. NCI had substantial programmatic involvement and actively supported awardees throughout the initiative. While awardees maintained custody and primary rights to data and software in accordance with governmental policies, the NCI retained full access to recruitment data, course materials, and evaluation metrics. An NCI Program Officer was responsible for the scientific and programmatic stewardship of the awards, and several NCI staff members were designated as NASDC Project Scientists and members of the SC, who collectively held one vote. These Project Scientists were instrumental in identifying eligible participants and scaling the initiative nationally.

### NASDC Clientele

Eligible participants were junior faculty investigators who had already received NCI awards, including career development (K), R00, R21, and first R01-equivalent grants. The pool was initially estimated at 1500 recipients. This clientele should not be confused with the NIH-defined category of early-career investigator (ESI), which is determined by the number of years since the completion of a terminal research degree or the end of postgraduate clinical training. NCI grantees of interest for NASDC were at a critical juncture in their research careers and were expected to compete successfully for additional NIH funding to become academic leaders teaching, mentoring, and managing research programs in a hypercompetitive funding climate.

### Course Settings

The first course started in year 1, and two courses were offered in both years 2 and 3. Two UE5 sites gave an additional course in year 3. The courses were free of charge for all participants. All instructional activities for each course had to be completed within 6 months. Each course was evaluated by participants four times via surveys, including pre-course, immediately post-course, 6 months post-course, and 12 months post-course. Per the funding guidelines, courses were required to offer in-person educational activities. With the onset of the COVID-19 pandemic, this could not be implemented; all courses were fully virtual for cohorts 1–3. Some sites delivered in-person teaching for cohorts 4 and 5. The consortium did not generate any data to compare in-person versus virtual teaching methods [3].

## Results and Discussion

### Coordinating Center Infrastructure Business Techniques

**Fig. 2:**
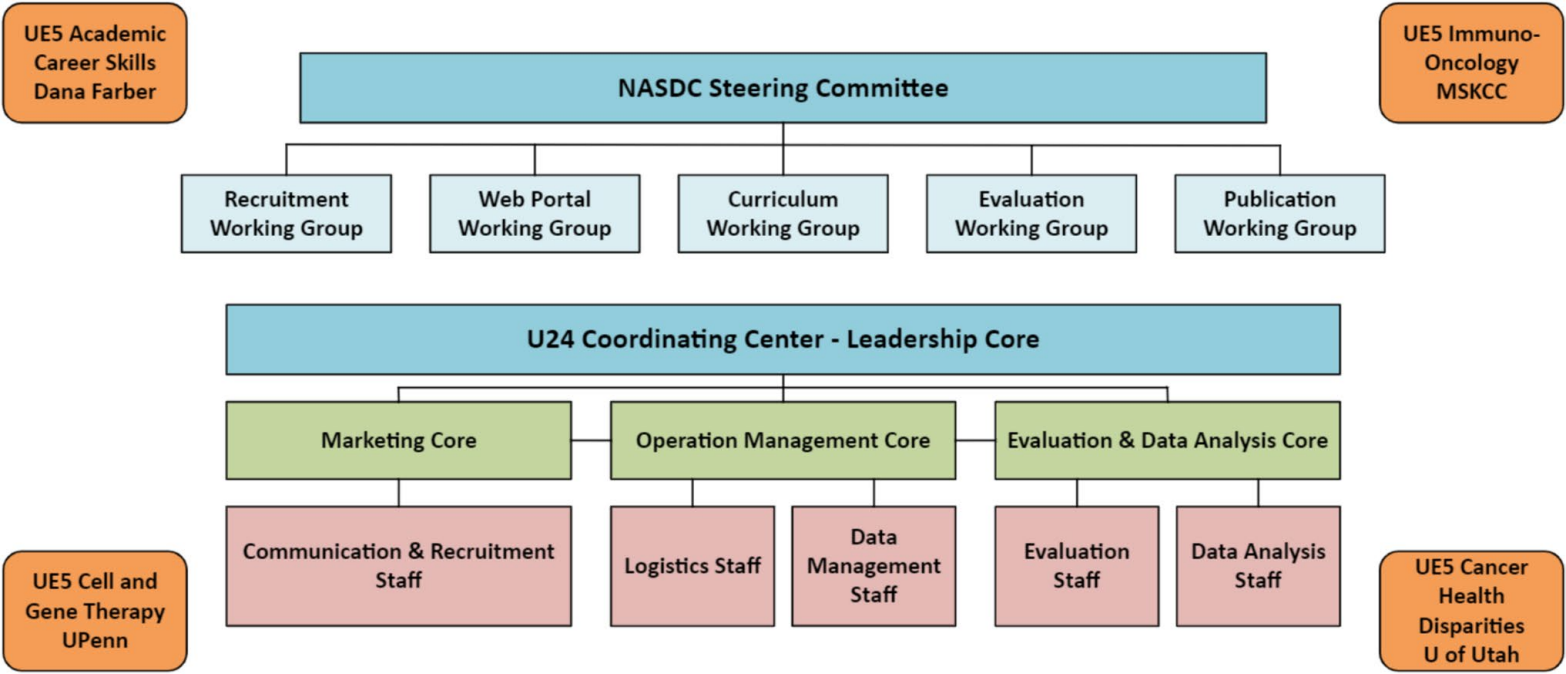
NASDC organization

**Fig. 3:**
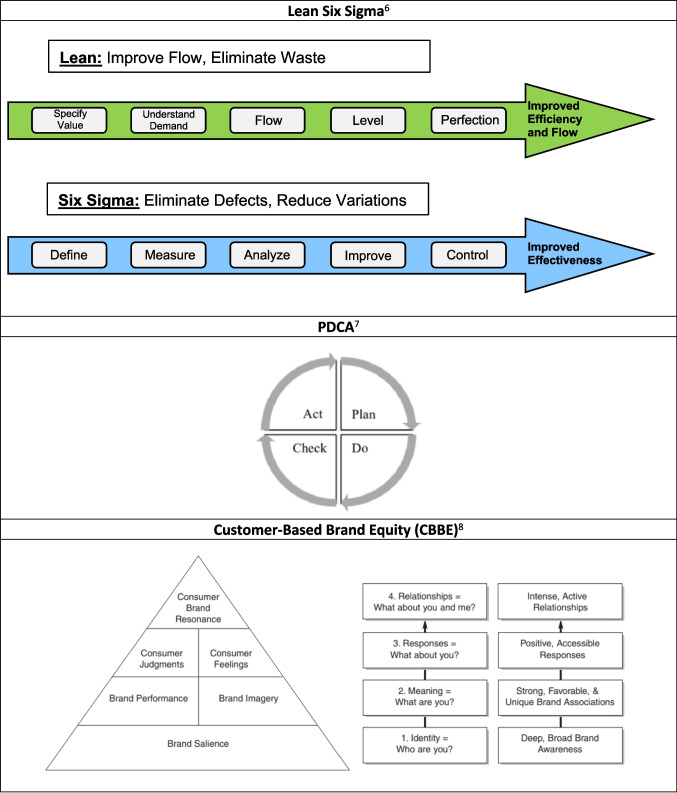
Business tools used by NASDC

The U24 CC was organized into four complementary cores that formed the framework for NASDC activities: (1) Leadership Core, (2) Evaluation and Data Analysis Core, (3) Operation Management Core, and (4) Marketing Core (Fig. 2). Table 1 details the administrative tasks and efficiency models used to build the NASDC infrastructure. Modern business techniques were applied for operational excellence: Lean is an efficiency approach focused on improving the material and information flow and reducing waste, whereas Six Sigma is an effectiveness approach focused on reducing variations. The assumptions are that waste removal will improve performance, and that system output improves if variations within processes are reduced. The main goal is to reduce flow time through a uniform process output (Fig. 3) [4]. Hence, the Lean Six Sigma management approach is a strategy that enhances the quality and effectiveness of education and infrastructure support for the implementation of research projects [5]. Plan-Do-Check-Act (PDCA) [6] is a results-based, adaptive model to improve efficiency [7]. These business excellence techniques sustained NASDC’s high-quality and efficient operations. To engage participants in NASDC initiatives and retain them in cancer research throughout long and productive academic careers, a multipronged communication strategy was used based on Keller’s brand equity model [8]. There are four levels of brand development in Keller’s Brand Equity Model with corresponding objectives at each stage. Brand equity helps people connect with the consortium.

**Table 1:** Staff—NASDC Coordinating Center

Staff	Core	Role
Lean Six Sigma operations manager	Leadership	Works closely with PIs and co-I; supervises the infrastructure, hiring, development of workflows and SOPs, and day-to-day administration oversight of all NASDC activities
Marketing manager	Marketing	Develops the marketing strategy and communication plan; works closely with the Operations Management Core
Communication specialist	Marketing	Ensures recruitment of junior faculty NCI awardees (clientele) and supports NASDC relationships
Web master	Operations management	Develops and maintains the NASDC portal and ensures appropriate data collection
Program coordinator	Operations management	Provides logistics support, including travel and meeting support
Evaluation specialist	Evaluation & data analysis	Develops the Kirkpatrick and the logic models, questionnaires, and scorecards; performs interviews for the evaluation of courses, participants, and the program
Data analyst	Evaluation & data analysis	Collects and analyzes survey data

### Coordinating Center Infrastructure Formation

The U24 CC was the administrative center of the NASDC infrastructure and supported the following activities drafted into a broad strategic plan:

1. Collaboration between UE5 and U24 investigators
2. Staffing the SC and WGs
3. Serving as a voting member on the NASDC SC
4. Adhering to the decisions and recommendations of the SC to the extent compatible with applicable grant regulations
5. Communications with NCI
6. Recruitment and onboarding of NASDC course participants
7. Adherence to the NASDC master course schedule
8. Developing program-specific evaluation instruments for participants and faculty
9. Collection of NASDC-specific evaluations
10. Curation of evaluation data
11. Participation in NASDC-specific analyses, presentations, and publications
12. Presentation of results to the SC and at professional conferences and supporting the publication of results
13. Ensuring that interactions with the UE5 s and U24 CC met the NASDC goals and objectives
14. Co-organizing and attending the only in-person NASDC meeting

#### Legal Requirements

Each UE5 and the U24 CC requested Institutional Review Board exemption 2 (45 CFR 46.104 (d)(2)), a process that took over 6 months. Data use agreements (DUAs) were signed between the U24 CC and the NCI and between the U24 CC and each UE5. This allowed each UE5 site to maintain propriety over the data it generated with an agreement to share data with the U24 for NASDC program data analysis. The DUAs between the U24 and each UE5 took between 5 and 12 months to be finalized and signed.

#### Steering Committee

The SC was created as NASDC’s main governing board. At the first NASDC meeting, members reviewed the criteria for SC formation, and each component (all UE5 s, the U24, and the NCI Project Scientists) chose a voting representative. SC meetings were open to all NASDC investigators. The U24 CC drafted bylaws, which were edited and approved by SC voting members. An SC Chair was elected. The committee met monthly; minutes were kept and available on the NASDC private web portal. The SC oversaw the implementation of the proposed short courses and the different NASDC working groups.

Overall NASDC performance was closely monitored to identify impediments to success and develop appropriate strategies to overcome problems. A good example was the need to move all courses to a virtual setting because of the COVID-19 pandemic. The SC tasked the WGs with creating the infrastructure for the various domains needed to deliver the courses efficiently. The SC approved emerging NASDC policies and procedures that supported these various domains. Logistics activities were also reviewed and approved by the SC, such as shared tools for disseminating information, a master schedule of NASDC course offerings, an agenda for the only face-to-face SC meeting that was organized post-pandemic, communications, productive interactions across the consortium, the promotion of collaborations on research education, a proposal for joint presentations, and publications by consortium members. The SC collaborated with the NCI Project Scientists to ensure that NASDC optimally leveraged existing NIH and NCI resources and programs.

### Working Group Creation

WGs with appropriate UE5 and U24 CC representation were established to address the specific domains of required activities, and bylaws were approved for each.

#### Recruitment Working Group

One of the first actions was to draft a course application form for the clientele. A simple electronic document was initially created in the secure web application Research Electronic Data Capture (REDCap) [4], a Health Insurance Portability and Accountability Act-compliant, web-based survey tool, to help collect demographics and educational information about the applicants. Application to a specific course might also have required specific data, such as an NIH biosketch. Redundancies in the requested data were simplified to collect the data only once. This information was used by each UE5 to triage and select applicants. Later, the form was added to the web portal to streamline the application process.

The U24 CC obtained a list of potential participants who met funding requirements for eligibility through an NCI Project Scientist search of internal NIH databases and the internet, a process more arduous than forecasted because of the various NIH and public databases that needed to be screened.

To promote NASDC, blast emails were sent nationwide, reaching cancer centers, organizational leaders, and academic institutions, including those serving underrepresented and minority populations. Social media was used to contact the NCI community; for each cohort, the courses were advertised on the @NCI_Training and @theNCI Twitter/X and LinkedIn accounts and amplified by individual UE5 s. To further increase reach, the associate directors for education at NCI-designated cancer centers were asked to send an invitation to their cancer center members. The NASDC consortium was also advertised at various national meetings.

Finally, a pre-course session was held about 1 month before course initiation to educate the participants on expectations and offerings, as well as to answer any questions.

#### Curriculum Working Group

The NASDC Curriculum WG had two focus areas: (1) review and disseminate UE5 short-course teaching methods and (2) share UE5 short-course curricula to enhance each UE5 short-course offering and leverage components that could be used across the courses.

Course delivery was virtual during the pandemic. The Curriculum WG supported efforts on innovative methodologies for video-based teaching. NASDC members contributed key components that included talks from course collaborators or colleagues at each center with relevant experience. Topics included the following:

- Tools for the delivery of virtual teaching and related materials (UPenn, OSU)
- Approaches to engage learners and encourage participation in video-based teaching (OSU, Harvard)
- Methods to create a community of teachers and learners for video-based teaching within each UE5 site and across sites (Harvard, Penn, Utah, MSK)

Sharing such information had a major role in successfully delivering excellent virtual short courses, which were favorably reviewed by most NASDC learners. Other innovations in remote course delivery included the “simulive” approach, where prerecorded lectures were supported online by the course instructor via Zoom chat and live Q&A following the lecture.

The Curriculum WG also focused on common elements needed across sites, such as responsible conduct of research content, content relevant to faculty development and advancement, and approaches to learner follow-up after the short course was completed. Curricula elements were exchanged between courses, particularly leveraging common elements between the two skill-based UE5 s (Harvard, Utah) and the more methodology-focused courses that had a common theme of immune- and cell-based therapy (MSK, Penn). Ways to enhance broad skills for cancer research and skills with a specific scientific focus were discussed at the meetings of this WG.

#### Portal Working Group

The Portal WG’s responsibilities were to create a NASDC website that provided access to registration and course materials for the learners and information for consortium members and the public. The portal seamlessly supported the conduct of activities and processes for NASDC. The portal infrastructure was created based on a software framework (built by the OSU Department of Research Information Technology) that provides:

- A public site to host information accessible to everyone, including the consortium description, course overview, participating UE5 s, leadership, WGs, and committees. The site also featured a calendar of upcoming key course dates, deadlines, and events to ensure all participants were informed about important timelines. In addition, the site included a dedicated social media page where users could access Twitter/X updates and engage with the course community
- Authentication for secure access to protect course content and evaluation data
- Access for individual users across the different UE5 groups and their corresponding courses. The site also provided updates on course policies, requirements, and expectations, ensuring that all participants were informed about prerequisites, attendance policies, and other important details
- Separated secure groups for each UE5 with access to its own data and some of the deidentified data collected through the evaluations. The combination of authentications and authorizations aligned to allow an end user to access the secure group
- A content management system to allow the U24 and UE5 s to add content to the portal across the various activities conducted in the NASDC consortium, which also serves as an archive for storing information and materials from past courses, organized by year
- An application management system enabling the automated management of course applicants’ information accessible by UE5 s to annotate decisions (accept/waitlist/deny/flag), with a notification system that allowed UE5 s and applicants to be aware of decisions and acceptance. The system also included the capability to export data, allowing for efficient saving of information, management of registered users and waitlist choices, and tracking of statistics related to applicants for further analysis and decision-making
- A testimonial page where past participants could share their experiences and feedback about the courses. This provided new applicants with valuable insights into the courses’ impact and helped foster a sense of community

The public web portal is available at https://nasdc.osu.edu/public/welcome.

#### Evaluation Working Group

**Fig. 4:**
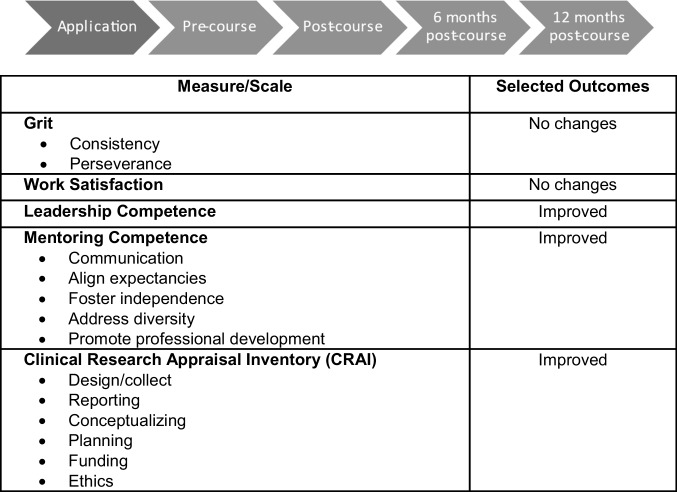
Evaluation process and outcome measures

Responsibilities of the Evaluation WG included the design of an integrative evaluation across the consortium, including identifying unique (course-specific) and shared metrics across workshop evaluations, the creation of a consortium-wide system for tracking trainee progress, and the identification of metrics for overall NASDC assessment ^5^. Evaluation forms were created through REDCap. Multiple serial evaluations collected longitudinal data. Continuing reassessment of each step was used to improve the infrastructure and led to the adoption of a web-based application form to facilitate records and access for the consortium members. Aspects of this education program that were assessed (Fig. 4) included:

1. Number of applications received to attend the course
2. Aggregate number and demographic and other characteristics of the course participants, including their career stage and types of prior NCI awards received
3. Pre- and post-course surveys of knowledge and skills acquired by participants
4. Participants’ feedback on the course and faculty/mentors
5. Faculty/mentors’ feedback on the course and participants
6. Short-term follow-up surveys of participants to assess the implementation of course content, grant applications submitted and awards received, new publications, and career advancements
7. A long-term assessment is planned to assess the power of the NASDC-provided education in promoting the participants’ careers. We plan to follow these participants throughout their academic careers through the use of public data repositories and/or working with Academic Analytics, a company OSU has contracted with

#### Publications Working Group

The Publications WG started in year 3, as the NASDC needed time to gather data. Its responsibilities included developing a strategic and coordinated plan for future publications, ensuring the quality of NASDC publications, reviewing manuscripts and abstracts submitted, and collecting participants’ publication data. A systematic process to submit abstracts and manuscripts was instituted to allow for review prior to publication and ensure coordination of NASDC publications.

### Participant Demographics

All applicants had to complete the standard application form. Application to more than one course was authorized. The application included standard demographics, background information on professional achievements, types of grants previously obtained, and field of study (Table 2). Eligible applicants were junior faculty who were program director/principal investigator (PD/PI) of a current NCI-funded grant (including NIH grant activity codes K01, K07, K08, K22, K23, K25, R00, R21, DP1, DP2, DP5, R01, R23, R29, R37, R56, RF1, RL1, U01). Each participant was selected by the UE5 leadership team for the corresponding course offering. The courses enrolled between 18 and 25 learners. Longitudinal evaluation surveys were required and will be reported in another paper in this NASDC series. A total of 305 junior faculty participated in one or more courses. About half were experts in public health; of the other half, one-third each specialized in basic science, translational science, or clinical science (Table 3). Most participants were K (42%) or R awardees (40%). Fifty-six (18%) had multiple awards, including 29 (10%) with both K or other, and R awards (Tables 4 and 5). Only 5% were multi-PIs.

**Table 2:** Eligibility criteria

**Demographics**	**Comments**
Full Name	
ORCID Number	Mandatory
**Professional Status**	
Affiliation	
Current Position	Academic Rank below Associate Professor
NCI Funding for the past 5 years	
Type of awards	K01, K07, K08, K22, K23, K25, R00, R01, R21, R23, R29, R37, R56, RF1, RL1, DP1, DP2, DP5, or U01
Current status of grant(s)	Active, No Cost Extension

**Table 3:** Disciplines Represented in NASDC

Frequencies of disciplines combo per participant (n = 305)		
Discipline	Frequency	%
Prevention and public health	143	46.9
Translational science	60	19.7
Basic science	49	16.1
Clinical science	41	13.4
Other and diagnosis	12	3.9
Total	305	100.0

**Table 4:** NCI Grant activity of NASDC participants: participant productivity

All participants are junior faculty PD/PI with current NCI funding: • 95% are PI • 5% are a Multi-PI		
# of awards/person	*N*	%
1 award	249	81.6
2 awards	43	14.1
> 2 awards	13	4.3
Total	305	100

**Table 5:** NCI Grant activity of NASDC participants: award types

Award(s)	All 5 cohorts—unique participants	
	#	%
K08	68	22.3
R01-equivalent	64	21.0
R21	32	10.5
K01	30	9.8
R00	25	8.2
K22	17	5.6
K07	12	3.9
K25	1	0.3
2 or 3 R awards	27	8.9
1–2 R awards, Other	9	3.0
1 K award, 1 R award	10	3.3
1 K award, Other	5	1.6
1 K award, 2–3 R awards	3	1.0
1 K award, 1 or more R award, Other	2	0.7
Total	305	100.0

### UE5 Courses

Four courses received UE5 awards to initiate the NASDC (Table 6). They will be detailed in the second paper of this series, which will analyze the experience of creating the online curriculum, teaching techniques, collegiality and collaboration, and commonalities such as responsible conduct of research.

**Table 6:** Calendar of courses

NASDC course details				
UE5	Course	Number of courses delivered	Dates	Format
Harvard/DFCI	Leadership skills	5	February 2021; September 2021; February 2022; September 2022; February 2023	Virtual
MSK	Immuno-oncology	6	March 2021; October 2021; March 2022; November 2022; January 2023; March 2023	Virtual
UPenn/CHOP	Cell therapy	5	February 2021; October 2021; February 2022; October 2022; February 2023	Virtual
Utah	Health disparities	3	February 2021; September 2021; March 2022	Virtual
		3	September 2022; February 2023; September 2023	In person

1. *Academic Career Skills: Leadership, Collaboration, and Resilience* at the Dana Farber Cancer Institute (DFCI)/Massachusetts General Hospital Cancer Center
2. *Immuno-Oncology for the Translational Researcher Short Course* (ITRSC) at Memorial Sloan Kettering Cancer Center (MSK).
3. *The Cell and Gene Therapy Toolkit for Junior Faculty* at the University of Pennsylvania/Children’s Hospital of Philadelphia (UPenn/CHOP).
4. *Advanced Course on Cancer-Related Health Disparities Research, Mentoring, and Leadership* at the University of Utah/Huntsman Cancer Institute.

## Conclusion

Launched in 2020, the NASDC consortium comprised a U24 CC to manage logistics and evaluations for four innovative, state-of-the-art courses that delivered evidence-based scientific and educational content. The goals of NASDC were to equip junior faculty with leadership skills, advanced technological expertise, and confidence in research. NASDC participants met the criteria for a successful career in a variety of domains. Participants applied for these courses through the NASDC portal (https://nasdc.osu.edu/public/welcome). Due to the COVID-19 pandemic, all courses transitioned to a virtual format. Course evaluations are ongoing, with outcomes to be reported after data curation. The long-term success of junior faculty grantees will be further benchmarked against publicly available national metrics. NASDC’s long-term goal is to cultivate a pool of innovative and expert cancer researchers who will advance patient care and improve health outcomes.

## Data Availability

The data underlying this article is available in the article and in its online supplementary material.

## Appendix

Table 7

**Table 7:** NASDC Consortium Members and CREDIT Contributions (nasdcsupport@osumc.edu)

Alphabetical list of authors	Institution	Email	Manuscript contributions
Anyidoho, Abena PhD	The Ohio State University Comprehensive Cancer Center	anyidoho.1@osu.edu	Investigation, data curation
Callahan, Margaret	University of Connecticut (formerly at Memorial Sloan Kettering Cancer Center)	mcallahan@uchc.edu	Conceptualization, writing (review and editing), supervision, project administration, funding acquisition
Eljanne, Mariam PhD	National Cancer Institute	mariam.eljanne@nih.gov	Resources, writing (review and editing), supervision, project administration
Fesnak, Andrew MD	University of Pennsylvania	fesnak@pennmedicine.upenn.edu	Writing (review and editing), project administration
Gray, Tamryn Fowler PhD, RN, MPH	University of North Carolina at Chapel Hill (formerly Dana-Farber Cancer Institute)	fowlert@unc.edu	Conceptualization, writing (review and editing), supervision, project administration, funding acquisition
Greer, Joseph PhD	Massachusetts General Hospital Cancer Center	JGREER2@mgh.harvard.edu	Conceptualization, writing (review and editing), supervision, project administration, funding acquisition
Hashibe, Mia PhD	University of Utah	mia.hashibe@utah.edu	Conceptualization, writing (review and editing), supervision, project administration, funding acquisition
Hexner, Elizabeth Olson MD, MSTR	University of Pennsylvania	EHexner@pennmedicine.upenn.edu	Conceptualization, writing (review and editing), supervision, project administration, funding acquisition
Jackson, Rebecca MD^a^	The Ohio State University Comprehensive Cancer Center	n/a	Conceptualization, methodology, project administration
Jedidi, Iness PhD	The Ohio State University Comprehensive Cancer Center	iness.jedidi@osumc.edu	Supervision, project administration, funding acquisition
Korczak, Jeannette PhD^b^	National Cancer Institute	n/a	Conceptualization, methodology, resources, writing (review and editing), supervision, project administration, funding acquisition
Lei, Ming	University of West Virginia (formerly at NCI)	ming.lei@hsc.wvu.edu	Conceptualization, writing (review and editing)
Mankoff, David MD	University of Pennsylvania	David.Mankoff@pennmedicine.upenn.edu	Conceptualization, writing (review and editing), supervision, project administration, funding acquisition
Mathur, Puneet MS, MBA	College of Medicine Research Information Technology, The Ohio State University	Puneet.Mathur@osumc.edu	Resources, project administration
Matthews, Lindsay MA	The Ohio State University Comprehensive Cancer Center (Graduated)	matthews.177@buckeyemail.osu.edu	Investigation, data curation
McMahon, Alyson BA	The Ohio State University Comprehensive Cancer Center	alyson.mcmahon@osumc.edu	Investigation, resources, writing (review and editing), visualization
Neill, Ushma S. PhD	Memorial Sloan Kettering Cancer Center	neillu@mskcc.org	Writing (review and editing)
Noël, Cauleen PhD	University of Pennsylvania	Cauleen.Noel@Pennmedicine.upenn.edu	Methodology, resources, writing (review and editing)
O’Connell, Ann EdD	Rutgers (formerly at The Ohio State University Comprehensive Cancer Center)	ann.oconnell@gse.rutgers.edu	Conceptualization, methodology, software, validation, formal analysis, investigation, resources, data curation, writing (review and editing)
Okuyemi, Kolawole MD, MPH	Indiana University (formerly at University of Utah)	kokuyemi@iu.edu	Conceptualization, writing (review and editing), supervision, project administration, funding acquisition
Pirl, William MD, MPH	Dana-Farber Cancer Institute	william_pirl@dfci.harvard.edu	Conceptualization, writing (review and editing), supervision, project administration, funding acquisition
Rodriguez, Romina MPA	Cornell University (formerly at Memorial Sloan Kettering Cancer Center)	rod2020@med.cornell.edu	Methodology, resources, writing (review and editing)
Tanner, Quinn MSPH	University of Utah	quinn.tanner@utah.edu	Writing (review and editing), project administration
Verschraegen, Claire MS, MD	The Ohio State University Comprehensive Cancer Center	claire.verschraegen@osumc.edu	Conceptualization, methodology, software, validation, formal analysis, investigation, resources, data curation, writing (original draft preparation), writing (review and editing), visualization, supervision, project administration, funding acquisition
Yusufov, Miryam PhD	Dana-Farber Cancer Institute	Miryam_Yusufov@dfci.harvard.edu	Conceptualization, writing (review and editing), supervision, project administration, funding acquisition
Zahir, Nastaran PhD	National Cancer Institute	nas.zahir@nih.gov	Resources, writing (review and editing), supervision, project administration

^a^Deceased

^b^Retired
